# Disseminated Coccidioidomycosis Presenting as Fever of Unknown Origin and Erythema Nodosum in a 3-Year-Old Child

**DOI:** 10.1155/2021/1755163

**Published:** 2021-10-15

**Authors:** Lauren Mills, Melanie M. Randall

**Affiliations:** Loma Linda University Medical Center, 11234 Anderson Street, Room A890A, Loma Linda, CA 92354, USA

## Abstract

Disseminated coccidioidomycosis is a disease caused by *Coccidioides* species, fungi endemic to the southwestern United States. We present here an uncommon case of a young child with erythema nodosum and fever of unknown origin, found to have the infection. While more common in adults, coccidioidomycosis should be considered in all patients with erythema nodosum.

## 1. Introduction

Coccidioidomycosis, also known as “San Joaquin fever” or “Valley fever,” is an infection caused by *Coccidioides* species, dimorphic fungi typically found in the soil [1]. The fungi are endemic to the southwestern United States [1]. Infections can be pulmonary or disseminated, with disseminated disease defined as skin, bone, joint, or meningeal involvement [2]. Patient populations at increased risk for disseminated or severe infection include African Americans, pregnant women, lower income individuals, and immunocompromised patients [2, 3]. Disseminated disease is much more common in adults than children and typically occurs several weeks to months after the initial infection [4]. Diagnosis can be confirmed with *Coccidioides* complement-fixing antibody titers of 1 : 16 or greater with evidence of active extrathoracic lesions [2]. *Coccidioides* infection has been shown to be on the differential for fever of unknown origin. Using the revised Durack definition of fever of unknown origin defined as temperature greater than 38.3 C, duration of greater than 3 weeks, and evaluation of at least 3 outpatient visits or 3 days in hospital, our patient was found to meet criteria [5]. Furthermore, our patient can be categorized into the classic fever of unknown origin category as fevers occurred secondary to infection [5]. Here, we present a rare case of a young child presenting with erythema nodosum and fever of unknown origin, found to have disseminated coccidioidomycosis.

## 2. Case Presentation

**Figure 1:**
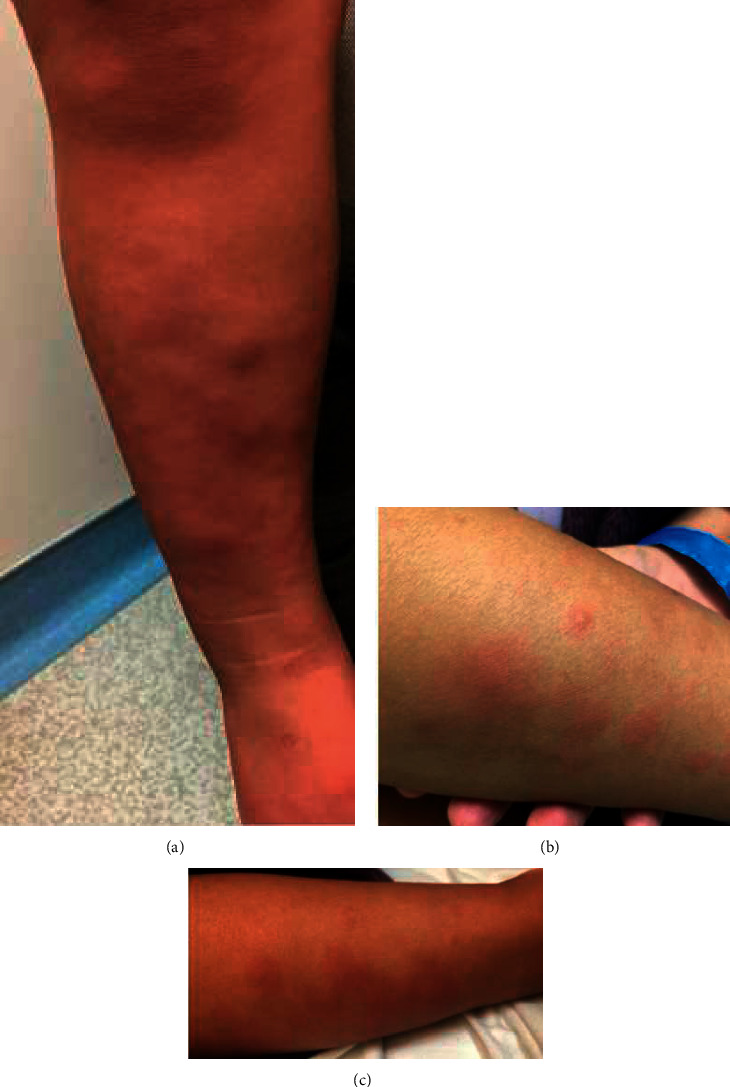
Photographs of the rash on the initial emergency department visit.

A 3-year-old female with no significant medical history presented to our emergency department (ED) with rash and fever. The onset of illness was three weeks prior with initial fever, cough, decreased oral intake, and emesis. These symptoms lasted two days and then resolved except for nightly fevers that persisted for additional 18 days. The patient developed a rash on day nine of the illness that lasted for five days. The rash was located on the bilateral upper and lower extremities, sparing the trunk and face. Both the fever and rash resolved for three days and then returned one day prior to ED presentation. On exam, the rash was erythematous, blanching, palpable, and tender nodules, most notable on the upper and lower extremities (Figure 1).

The vital signs were temperature 37.2 C, pulse 126 beats per minute, blood pressure 110/57 mm Hg, and oxygen saturation 100%. Labs were significant for a white blood cell count of 15,400/*μ*L (reference range 4,800–11,800), C-reactive protein of 7.7 mg/dL (reference range 0–0.8), and erythrocyte sedimentation rate of 50 mm/hr (reference range 0–20). A diagnosis of erythema nodosum (EN) was made. The patient was well appearing and tolerated oral intake, so she was discharged with referral to the outpatient infectious disease and rheumatology clinic.

The patient returned two days later with continued fever and worsening rash. She was admitted to the hospital for an infectious and rheumatologic evaluation. The initial workup was notable for negative blood and urine cultures, respiratory viral panel, and SARS-CoV-2 testing. However, the *Coccidioides* antibody titers were positive at 1 : 32. Testing for other infectious etiologies including *Histoplasma, Toxoplasmosis, Cytomegalovirus, Blastomyces, Epstein-Barr Virus, Mycoplasma*, and *Mycobacterium* was negative. Biopsy of the rash showed mild superficial perivascular dermatitis, deemed nonspecific. Rheumatologic workup consisted of additional laboratory testing including complement levels and an echocardiogram. Complement levels were not consistent with a rheumatologic process, and echocardiogram was unremarkable. A diagnosis of disseminated coccidioidomycosis was made, and the patient was started on fluconazole, with a plan to treat for 12 months. She was discharged home two days later with infectious disease follow-up. The patient only took a few days of fluconazole at home, as she did not tolerate the medication without emesis. Upon follow-up, the patient's symptoms had resolved with no more fevers or rash, and inflammatory markers had normalized. Given the patient's clinical improvement, the decision was made to monitor symptoms without reinitiating antifungal treatment. As patient was noted to improve without adequate treatment, it was thought that her own immune system was able to clear the infection.

## 3. Discussion

Our case illustrates a less common presentation of disseminated coccidioidomycosis in a 3-year-old girl. In multiple surveys and studies, patients less than 10 years old are the least commonly diagnosed with the infection [6–8]. She did, however, present with the classic rash of EN. EN is an acute nodular panniculitis that consists of firm, tender nodules, most commonly on the extensor surfaces of the leg and arms [9]. There are numerous infectious and rheumatologic etiologies of EN, with some studies showing coccidioidomycosis to account for up to 95% of EN cases [4, 10]. For patients living in endemic regions, coccidiomycosis should be considered in patients with fever of unknown origin or erythema nodosum [11–16].

## Data Availability

The data used to support the findings of this study are available from authors upon request (mrandall@llu.edu).
